# Air pollution and its impacts on health in Vitoria, Espirito Santo, Brazil

**DOI:** 10.1590/S1518-8787.2016050005909

**Published:** 2016-03-01

**Authors:** Clarice Umbelino de Freitas, Antonio Ponce de Leon, Washington Juger, Nelson Gouveia

**Affiliations:** I Laboratório de Investigação Médica. Hospital das Clínicas. Faculdade de Medicina. Universidade de São Paulo. São Paulo, SP, Brasil; II Instituto de Medicina Social. Universidade Estadual do Rio de Janeiro. Rio de Janeiro, RJ, Brasil; III Departamento de Medicina Preventiva. Faculdade de Medicina. Universidade de São Paulo. São Paulo, SP, Brasil

**Keywords:** Child, Adult, Air Pollution, adverse effects, Respiratory Tract Diseases, epidemiology, Cardiovascular Diseases, epidemiology, Time Series Studies

## Abstract

**OBJECTIVE:**

To analyze the impact of air pollution on respiratory and cardiovascular morbidity of children and adults in the city of Vitoria, state of Espirito Santo.

**METHODS:**

A study was carried out using time-series models via Poisson regression from hospitalization and pollutant data in Vitoria, ES, Southeastern Brazil, from 2001 to 2006. Fine particulate matter (PM_10_), sulfur dioxide (SO_2_), and ozone (O_3_) were tested as independent variables in simple and cumulative lags of up to five days. Temperature, humidity and variables indicating weekdays and city holidays were added as control variables in the models.

**RESULTS:**

For each increment of 10 µg/m^3^ of the pollutants PM_10_, SO_2_, and O_3_, the percentage of relative risk (%RR) for hospitalizations due to total respiratory diseases increased 9.67 (95%CI 11.84-7.54), 6.98 (95%CI 9.98-4.17) and 1.93 (95%CI 2.95-0.93), respectively. We found %RR = 6.60 (95%CI 9.53-3.75), %RR = 5.19 (95%CI 9.01-1.5), and %RR = 3.68 (95%CI 5.07-2.31) for respiratory diseases in children under the age of five years for PM_10_, SO_2_, and O_3_, respectively. Cardiovascular diseases showed a significant relationship with O_3_, with %RR = 2.11 (95%CI 3.18-1.06).

**CONCLUSIONS:**

Respiratory diseases presented a stronger and more consistent relationship with the pollutants researched in Vitoria. A better dose-response relationship was observed when using cumulative lags in polynomial distributed lag models.

## INTRODUCTION

The effects of air pollution on health are detected in several cities around the world. This led the World Health Organization (WHO) to propose reduction targets from decreasing Air Quality Guidelines, replacing the previous regulations, in force until 2005^16^. Studies on the impact of air pollution on health in Latin American countries yield results similar to those in other locations in the world^12^. Most studies refers to air pollutants resulting primarily or secondarily from burning fossil fuels: fine particulate matter (PM_10_), sulfur dioxide (SO_2_), carbon monoxide (CO), nitrogen oxides (NO_x_), and ozone (O_3_). As for health outcomes, respiratory and cardiovascular diseases are the ones most commonly associated with air pollution^16^.

**Figure 1:**
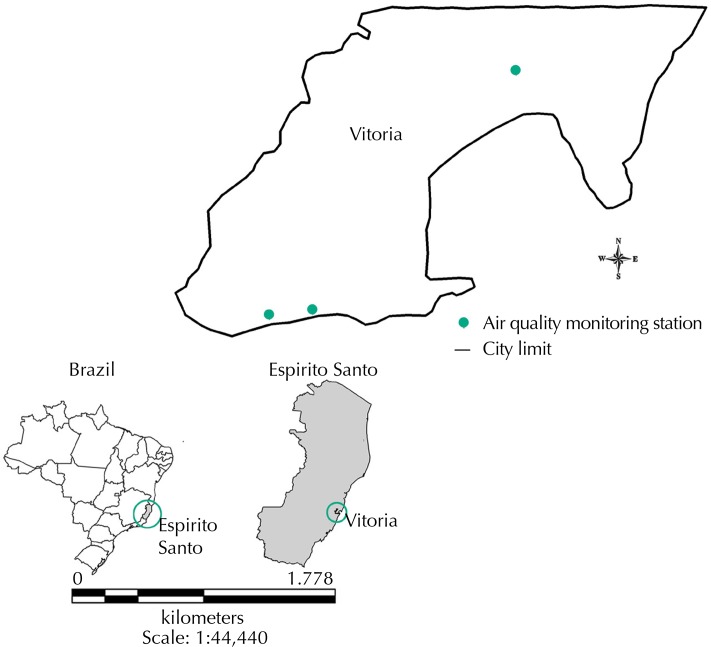
Air quality monitoring stations. Vitoria, ES, Southeastern Brazil.

Vitoria is part of a geographical area of great urbanization called Greater Vitoria metropolitan area. It is the fourth most populous city of the Espirito Santo state (327,801 inhabitants in a 98 km^2^ area) according to data collected by the Brazilian Institute of Geography and Statistics (IBGE) in 2010. It is surrounded by a river island called Vitoria Bay. It has two ports that are a part of the largest port complex in Brazil (Port of Vitoria and Port of Tubarao). Since 2000, the city has had an *Rede Automática de Monitoramento da Qualidade do Ar* (RAMQAR – Automatic Air Quality Monitoring Network ). This RAMQAR has three stations (Figure 1) and measures the parameters of PM_10_, SO_2_, O_3_, CO, and hydrocarbons in the neighborhoods of Jardim Camburi, Enseada do Sua, and Vitoria Centro^a^. Carapina station, located in a municipality close to Vitoria, monitors the meteorological parameters, in addition to measuring PM_10._

Large industrial projects were implemented in the Greater Vitoria Region since the early 1970s, and industry was identified as the main source of pollution in the area^a^. According to the same source, the growth of car fleet and real-estate projects has changed the area profile in recent years. An inventory of sources carried out by the *Instituto Estadual de Meio Ambiente e Recursos Hídricos* (IEMA – Institute for Environment and Water Resources of Espirito Santo) in 2011 indicates dust resuspension (68.0%), which has various origins, industries (21.0%), and vehicles (4.0%) as the main causes for PM_10_ levels. Industrial emissions accounted for almost all SO_2_ emission to the atmosphere and for more than half of NO_x_ emission, and a small relative share for hydrocarbon emission. In turn, vehicles were the main responsible for emitting CO and hydrocarbons (around 50.0%)^b^. Data collected by the IBGE Cities website in 2005 indicate an 83,859 car fleet, with an increasing trend, reaching 124,534 in 2014 of a total of 191,143 vehicles in all modes^c^.

Publications on the effects of air pollution in several Brazilian cities use different approaches, which has prevented comparisons between findings. Aiming at contributing to this comparison, the methodology used in this study was applied to various Brazilian cities that have air quality monitoring. This study aimed at estimating the risk of illness due to respiratory and cardiovascular diseases and its relation to air pollution in Vitoria.

## METHODS

Data of hospital admissions according to place of residence were obtained in the Hospital Information System (HIS) of DATASUS from 2001 to 2006 for the city of Vitoria, from an information website created for the *Projeto de Avaliação de Impacto da Poluição do Ar nas Cidades Brasileiras* (Air Pollution Impact in Brazilian Cities Assessment Project). The project aimed at measuring the impact of air pollution on health, using the same methodology for various locations, and exploring environmental monitoring models.

The website refers to the *Autorizações de Internação Hospitalar* (AIH – Hospital Admission Authorizations) systematized by the Department of Computer Science of the Brazilian Unified Health System (SUS), obtained from DATASUS. The bases were grouped per day, i.e., daily counts of hospital admissions due to the investigated causes: respiratory diseases (ICD10: J00-J99) at all ages (TRD), respiratory diseases (ICD10: J00-J99) in children under the age of five years (CRD), and cardiovascular diseases (ICD10: I00-I99) in adults over 39 years old (CVD), used as dependent variables in the models. The pollutant variables were grouped according to monitoring stations and meteorological variables in the same website. Meteorological and pollutant data from three monitoring stations in the city of Vitoria, provided by IEMA, were grouped in daily averages for PM_10_ and SO_2_, and the largest 8h-concentration for O_3_, minimizing losses in case the stations were considered individually. The bases were evaluated for information continuity, accepting losses of up to 15.0% of days in the period for each environmental variable. The concentration values of PM_10_, O_3_, and SO_2_ were used as exposure variables, and temperature and relative humidity as controls.

Using the data provided by the website as a source of information, a time-series analysis was performed with the *ares* library developed for the R application^d^, using the same methodology applied in cities that have joined the project. For each participating municipality, explanatory models were created for the counts of hospital admissions due to causes studied over time. The proposed models are part of the class of Generalized Additive Models (GAM), with the option of Poisson regression. In this class of models, the average daily number of health events was described as the sum of functions of explanatory variables. The terms whose association with the outcome was linear, e.g., weekdays and holidays, were included in the model multiplied by a slope. Variables that were nonlinearly related to the outcome were included in the model by smooth functions, e.g., splines, according to the equation:


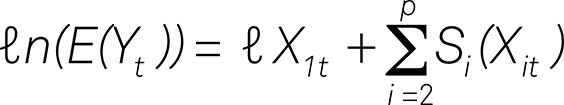


in which *Y*_t_ and *X*_1t_ are the numbers of morbid events and the level of a given pollutant on day *t*, respectively; *X*_*it*_ are the predictor variables, including time, and *S*_*i*_ are the smoothing functions, using natural splines. When indicator variables for weekdays and national or local holidays were added, its significance was tested. Holidays with significance of up to 0.09 were grouped according to the direction of the effect: positive or negative. In the time-series modeling process, the aim was to minimize the Akaike Information Criterion (AIC) and optimize the Partial Autocorrelation Function (PACF).

After building the working model (Core Model) containing all control variables and checking their suitability, pollutants in lags of up to five days (simple lag) were added individually. The cumulative effect in that period was also analyzed from a polynomial distributed lag model. This model, in addition to considering the latency of the effect of pollutants, minimizes the instability in the estimation process, typical of analyses that use multiple lags^13^.

The effect was estimated for each pollutant linearly added to the working model, providing the percentage of relative risk (%RR) for each increment of 10 µg/m^3^. This is derived from RR using the following formula: %RR = (*e* ^10β^ - 1)*100. This amount expresses the percentage change in the average number of hospital admissions for a variation of 10 µg/m^3^ in pollutant concentration. A 5% significance level was assumed in all estimates.

## RESULTS

We found a smaller number of child admissions, with an average of two per day due to respiratory diseases. Losses of meteorological and pollutant data were below 15.0% from January 2001 to December 2006, a limit established as acceptable for the period (Table 1).

**Table 1:** Basic descriptive parameters. Vitoria, ES, Southeastern Brazil, 2001-2006.

Parameter	Number of days with data	na	Average	SD	min	max	p_25_	p_50_	p_75_
Total respiratory diseases	2,191	0	3.9	2.4	0	15.0	2.0	4.0	5.0
Respiratory diseases in children < 5 years	2,191	0	2.1	1.7	0	10.0	1.0	2.0	3.0
Cardiovascular diseases in adults > 39 years	2,191	0	3.7	2.5	0	15.0	2.0	30	5.0
PM_10_	2,164	27.0	27.1	7.0	6.5	61.0	22.5	26.67	31.0
O_3_	1,917	274.0	45.2	17.3	0	119.0	33.0	44.0	56.0
SO_2_	2,154	370	11.7	5.5	1.0	54.0	8.0	11.0	15.0
T (°C)	2,166	25.0	24.4	2.5	16.8	30.4	22.48	24.4	26.4
RH (%)	2,157	34.0	78.8	5.8	57.8	95.5	74.8	78.6	82.6

na: number of days without information; PM_10_: particulate matter smaller than 10µ; O_3_: ozone; SO_2_: sulfur dioxide; T: temperature in degrees Celsius; RH: relative humidity

PM_10_: particulate matter smaller than 10μ; O_3_: ozone; SO_2_: sulfur dioxide

**Figure 2:**
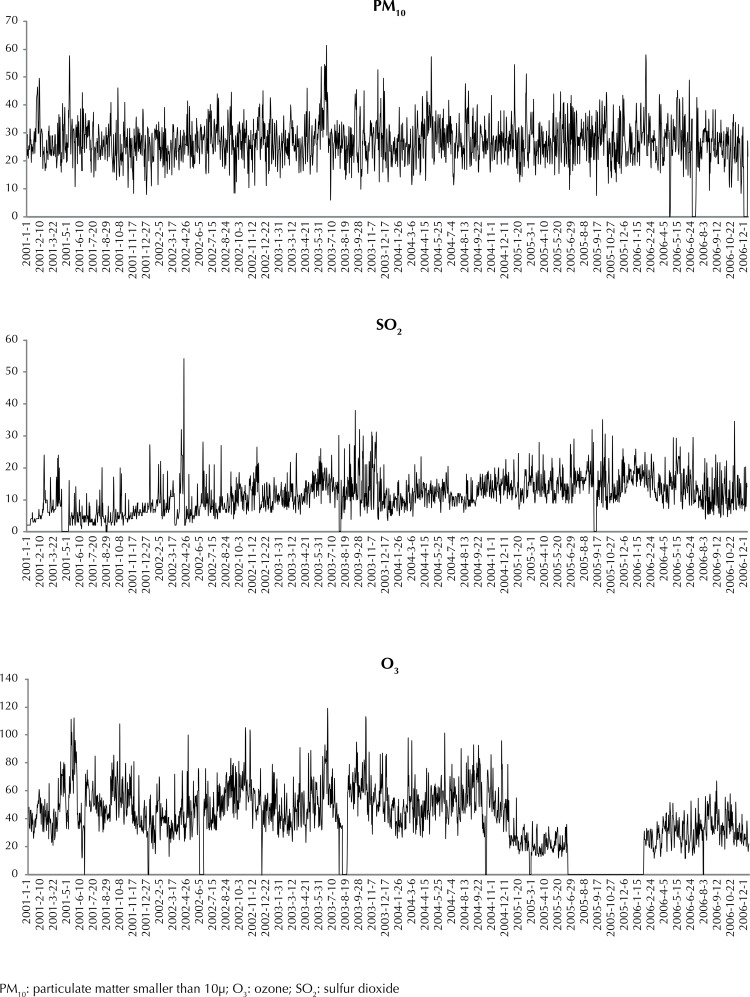
Daily pollutant levels (PM10, SO2, and Ozone). Vitoria, ES, Southeastern Brazil, 2001-2006.

Despite the characteristic seasonality of pollutants, annual PM_10_ averages remained constant and around 27 µg/m^3^ in the period. SO_2_ showed an increasing trend, with levels ranging from 5.87 µg/m^3^ in 2001 to 14.06 µg/m^3^ in 2006. Ozone data showed discontinuity between 2005 and 2006 because of problems in the monitoring station, not allowing trend observation (Figure 2).

Residue analysis indicated a good adjustment of the model for researched outcomes. Even with the small number of hospital admissions due to respiratory diseases in children under the age of five years, the distribution of standardized residues compared with the normal distribution in quantiles was similar to the distribution for cardiovascular and all respiratory diseases.

Upon adding pollutants in simple lag in the models, a significant relationship was found for PM_10_ at lag 0 with %RR = 4.40 (95%CI 0.64-8.23) when analyzing TRD. A relationship was found at lag 2 for CRD: %RR = 2.55 (95%CI 0.08-5.08) also for O_3_. Cardiovascular diseases were not related to any of the pollutants studied in this lag structure. Table 2 presents %RR of the polynomial distributed lag model from zero to five days for increments of 10 µg/m^3^ in pollutant levels and their overall value. The overall value reached high levels in the period for hospital admissions due to total respiratory diseases and in children under the age of five years. CVD were related only to ozone.

**Table 2:** Percentage of relative risk* and 95% confidence interval for hospital admissions due to total respiratory diseases. respiratory diseases in children under the age of five years. and cardiovascular diseases in adults over 39 years old. Vitoria. ES. Southeastern Brazil. 2001-2006.

Pollutant	Lag	TRD			CRD < 5 years			CVD > 39 years		
		
		%RR	95%CI		%RR	95%CI		%RR	95%CI	
PM_10_	Current day	1.52	-1.85	5.02	0.27	-4.34	5.09	-1.55	-4.87	1.88
	1-day lag	2.11	0.12	4.13	1.43	-1.28	4.22	-0.14	-2.12	1.88
	2-day lag	2.26	0.37	4.18	1.97	-0.62	4.62	0.66	-1.23	2.6
	2-day lag	1.98	0.15	3.85	1.87	-0.65	4.45	0.84	-1.02	2.75
	2-day lag	1.28	-0.43	3.03	1.13	-1.23	3.56	0.39	-1.39	2.2
	2-day lag	0.16	-2.74	3.16	-0.22	-4.23	3.95	-0.68	-3.62	2.35
	Cumulative 5 days	9.67	7.54	11.84	6.60	3.75	9.53	-0.49	-2.46	1.52
O_3_	Current day	-0.50	-2.32	1.34	0.33	-2.13	2.86	1.03	-0.88	2.98
	1-day lag	0.08	-0.91	1.07	0.84	-0.49	2.2	0.26	-0.77	1.3
	2-day lag	0.47	-0.49	1.43	1.06	-0.23	2.36	-0.14	-1.13	0.87
	2-day lag	0.67	-0.27	1.63	0.96	-0.32	2.26	-0.16	-1.14	0.83
	2-day lag	0.69	-0.24	1.63	0.57	-0.68	1.83	0.19	-0.76	1.16
	2-day lag	0.52	-1.19	2.25	-0.13	-2.41	2.21	0.92	-0.86	2.73
	Cumulative 5 days	1.93	0.93	2.95	3.68	2.31	5.07	2.11	1.06	3.18
SO_2_	Current day	1.36	-3.4	6.35	-0.25	-6.53	6.44	-5.21	-9.88	-0.31
	1-day lag	0.20	-2.44	2.91	0.55	-2.98	4.2	-2.1	-4.83	0.72
	2-day lag	-0.15	-2.74	2.5	1.08	-2.38	4.66	0.37	-2.39	3.2
	2-day lag	0.29	-2.29	2.94	1.34	-2.11	4.91	2.13	-0.65	4.98
	2-day lag	1.53	-0.97	4.09	1.33	-1.99	4.76	3.14	0.45	5.9
	2-day lag	3.61	-0.87	8.29	1.04	-4.81	7.25	3.38	-1.34	8.33
	Cumulative 5 days	6.98	4.17	9.88	5.19	1.5	9.01	1.42	-1.41	4.34

PM_10_: particulate matter smaller than 10µ; O_3_: ozone; SO_2_: sulfur dioxide; TRD: Hospital admissions due to total respiratory diseases; CRD < 5 years: Hospital admissions due to respiratory diseases in children under the age of five years; CVD > 39 years: Hospital admissions due to cardiovascular diseases in adults over 39 years old

* Expresses the percentage change in the average daily number of hospital admissions for a variation of 10 µg/m^3^ in the pollutant concentration.

## DISCUSSION

Health outcomes presented a more robust and consistent relationship when considering the cumulative effects of pollutants from zero to five days in Vitoria. Hospital admissions due to total respiratory diseases and respiratory diseases in children under the age of five years were related to all researched pollutants, most strongly associated with PM_10_. Admissions due to cardiovascular diseases were associated only to variations in ozone levels. The results expressed in %RR indicate how much the average daily number of admissions increases for each increment of 10 µg/m^3^ of the pollutant.

The PM_10_ levels (27 µg/m^3^) in Vitoria from 2001 to 2006 remained below the annual average of 50 µg/m^3^ of the air quality standards proposed by the Brazilian National Environmental Council (CONAMA), and close to the 20 µg/m^3^ recommended by WHO guidelines. Nevertheless, the effect of air pollution was found on residents of Vitoria. This supports the WHO recommendation to always seek lower exposure levels for health protection, even in cities of little pollution, as the dose-response relationship is linear when the effects of air pollutants are analyzed without a safe dose^4^ ^,^ ^16^. Vitoria has a mixed exposure characteristic (industry and vehicles contribute to pollutant levels) and must have a PM_10_ composition different from other urban centers where vehicles predominate. To understand the effects of each type of exposure source, speciation studies on particulate matter are required, but this is not available in the location.

Although a period of more data completion was considered for the study, gaps in the information on ozone levels were noted. These gaps, although within tolerable limits according to the criteria established by the authors, are found on a single period of 2005, which may have influenced the results of the analysis of this pollutant.

Among the pollutants, PM_10_ presents the most frequent and consistent relationship with cardiovascular and respiratory tract diseases, although it is not always possible to separate the effects of other pollutants, as they are all present in the air we breathe^1^. A review study on the mechanisms of action of PM_10_ indicates the onset of oxidative stress and the systemic inflammatory process that may be related to the anatomical and physiological lung remodeling and the atherosclerotic process^1^. We found a relationship between respiratory diseases and PM_10_ in Vitoria. This pollutant was not related to hospital admissions due to cardiovascular diseases.

Bell et al.^2^ performed a meta-analysis of time-series studies and found a relationship between ozone and overall mortality and mortality due to cardiovascular diseases considering American cities and other locations. Deaths from respiratory diseases were not associated with ozone levels. In a recent publication of 34 studies evaluating the effects of PM_10_, PM_2,5_, CO, NO_2_, SO_2_, and O_3_ on myocardial infarction, no significant relationship was found between the latter pollutant and this outcome^10^. Exposure to ozone was significantly associated to hospital admissions due to respiratory diseases in children^8^ ^,^ ^11^ and cardiovascular diseases in adults over 39 years old living in Cubatao, on the coast of the state of Sao Paulo^11^. In the city of Sao Paulo, this relationship was observed for respiratory diseases in children, with a weaker association than the one found in this study. In Sao Paulo, the risk of admissions was evaluated for increments of 10 µg/m^3^, which allowed the comparison with our findings^5^.

The literature shows that effects on health continue to be detected even in low exposure to sulfur dioxide^15^. This should become a greater concern in Vitoria, as an increasing trend of this pollutant was observed in the studied period. This trend persisted, as noted in the *Relatório Anual de Qualidade do Ar de Vitória de 2013* (2013 Annual Vitoria Air Quality Report), which analyzed the past 10 years.

In a literature survey on effects of air pollution on the health of residents of Vitoria, three publications were found. The first one^9^ explores the attributable proportion of deaths due to respiratory diseases in the elderly and children in the Brazilian urban population, including Vitoria, by the application of the coefficient of dose-response relationship in time-series studies performed in the cities of Rio de Janeiro and Sao Paulo. The second one^3^ analyzes the rates of outpatient care for asthma according to Vitoria neighborhoods. The third one^14^ explores time-series models – both those already established in the literature and principal component analysis. With the two approaches, the authors aim at establishing a dose-response relationship between pollutants and hospital admissions in children, indicating a better suitability of the model and the pollution impact when using principal component analysis. With this approach, %RR = 3.00 was found for the increment of 10.49 µg/m^3^ of PM_10_, while the %RR obtained from the conventional approach was 2.00 for the same pollutant increment. While it is difficult to compare when using different analysis methodology, this study found the highest estimate for children under the age of five years, with %RR = 6.6 for each increment of 10 µg/m^3^ of PM_10_, when considering the cumulative effect of zero to five days.

The AIH information system does not represent the totality of hospitalizations in Brazil. It refers to services paid by the Brazilian Unified Health System and its coverage varies among states. The information system of the *Comunicação de Internação Hospitalar* (CIH – Hospital Admission Communications), which has a record of admissions in hospitals not covered by SUS, is available on DATASUS from 2008 on. The CIH accounted for 16.9% of hospitalizations in Vitoria in 2008. Estimating from this proportion, this study may have used more than 80.0% of the hospitalizations in the city. However, interpreting the results requires caution. They should be considered as the impact of air pollution on admissions recorded in hospitals related to by SUS.

Studies aimed at measuring the impact of pollution on health are a tool used by Health Surveillance technicians to discuss prevention and control activities with regulatory agencies or the judiciary^6^. On the one hand, the health sector has little legal power to require measures to correct the contamination of the environment. On the other, it has instruments that allow measuring the damage caused by this contamination in the population, although these are complex measurement techniques that are not always accessible to primary care services.

The Brazilian Ministry of Health proposes assessing and monitoring damage to health caused by air pollution using time-series studies as a surveillance activity^7^. In a recent study^7^, PM_10_ was proposed as an exposure indicator and hospital admissions due to respiratory diseases as an effect indicator for the purposes of monitoring impacts of air pollution on health in Brazilian cities. Since the health sector does not have legal mechanisms for air pollution control, its contribution to the implementation of policies involves quantifying the impact of pollution on people’s disease burden. Although they only measure the acute effects of air pollution, time-series studies can be used to raise awareness of managers and the population, supporting the adoption of policies aimed at improving the air we breathe. The environmental area of Vitoria can use these studies to advance the air quality control policies in the city. Encouraging the continuity and diversity of approaches that aim at relating harsh environmental conditions to their impacts on health is not only desirable, but necessary for structuring Environmental Health in Brazil and Latin America as a whole.

## Footnotes

a Instituto Estadual de Meio Ambiente e Recursos Hídricos. Relatório da qualidade do ar: Grande Vitória: 2013 [Internet]. Vitória (ES): Instituto Estadual de Meio Ambiente e Recursos Hídricos; 2013 [cited 2014 Sept 14]. Available from: http://www.meioambiente.es.gov.br/download/Relat%C3%B3rio_Anual_de_Qualidade_do_Ar_2013.pdf

b Instituto Estadual de Meio Ambiente e Recursos Hídricos. Meio ambiente: estudo mostra como está a qualidade do ar na GV [Internet]. *Diário Oficial de Poderes de Estado*. Vitória (ES) 11 maio 2011, 3. [cited 2015 Nov 26]. Available from: http://www.dio.es.gov.br/portal/visualizacoes/pdf/#/p:1/e:241

c Instituto Brasileira de Geografia e Estatística, Cidades. Espírito Santo: Vitória. Rio de Janeiro: Instituto Brasileira de Geografia e Estatística; 2015 [cited 27 Nov 2015]. Available from: http://www.cidades.ibge.gov.br/xtras/perfil.php?lang=&codmun=320530

d Junger WL. Análise, imputação de dados e interfaces computacionais em estudos de séries temporais epidemiológicas [thesis]. Rio de Janeiro (RJ): Universidade Estadual do Rio de Janeiro; 2008.

## References

[1] Anderson JO, Thundiyil JG, Stolbach A (2012). Clearing the air: a review of the effects of particulate matter air pollution on human health. J Med Toxicol.

[2] Bell ML, Dominici F, Samet JM (2005). A meta-analysis of time-series studies of ozone and mortality with comparison to the national morbidity, mortality, and air pollution study. Epidemiology.

[3] Castro HA, Hacon S, Argento R, Junger WL, Mello CF, Castiglioni N (2007). Doenças respiratórias e poluição atmosférica no Município de Vitória, Espírito Santo, Brasil. Cad Saude Publica.

[4] Daniels MJ, Dominici F, Samet JM, Zeger SL (2000). Estimating particulate matter-mortality dose-response curves and threshold levels: an analysis of daily time-series for the 20 largest US cities. Am J Epidemiol.

[5] Freitas C, Bremner AS, Gouveia N, Pereira LA, Saldiva PH (2004). Internações e óbitos e sua relação com a poluição atmosférica em São Paulo, 1993-1997. Rev Saude Publica.

[6] Freitas CU, Campos RAG, Silva MAFR, Panachão MRI, Moraes JC, Waissmann W (2010). Can live in the surroudings of a petrochemical complex be a risk factor for autoimmune thiroid disease?. Environ Res.

[7] Freitas CU, Junger W, Leon AP, Grimaldi R, Silva MAFR, Gouveia N (2013). Poluição do ar em cidades brasileiras: selecionando indicadores de impacto na saúde para fins de vigilância. Epidemiol Serv Saude.

[8] Jasinski R, Pereira LA, Braga ALF (2011). Poluição atmosférica e internações hospitalares por doenças respiratórias e crianças e adolescentes em Cubatão, São Paulo, Brasil, entre 1997-2004. Cad Saude Publica.

[9] Marcilio I, Gouveia N (2007). Quantifying the impact of air pollution on the urban population of Brazil. Cad Saude Publica.

[10] Mustafić H, Jabre P, Caussin C, Murad MH, Escolano S, Tafflet M (2012). Main air pollutants and myocardial infarction: a systematic review and meta-analysis. JAMA.

[11] Nardocci AC, Freitas CU, Ponce de Leon ACM, Junger WL, Gouveia NC (2013). Poluição do ar e doenças respiratórias e cardiovasculares: estudo de séries temporais em Cubatão, São Paulo, Brasil. Cad Saude Publica.

[12] Romieu I, Gouveia N, Cifuentes LA, Ponce de Leon A, Junger W, Vera J (2012). Multicity study of air pollution and mortality in Latin America (the ESCALA study). Res Rep Health Eff Inst.

[13] Schwartz J (2000). The distributed lag between air pollution and daily deaths. Epidemiology.

[14] Souza JB, Reisen VA, Santos JM, Franco GC (2014). Componentes principais e modelagem linear generalizada na associação entre atendimento hospitalar e poluição do ar. Rev Saude Publica.

[15] Souza SL, Pires JC, Martins EM, Fortes JD, Alvim-Ferraz MC, Martins FG (2012). Short-term effects of air pollution on respiratory morbidity at Rio de Janeiro – Part II: health assessment. Environ Int.

[16] World Health Organization (WHO) (2005). Air quality guidelines: global update 2005: report on a.

