# ERRATA

**DOI:** 10.1590/S01518-8787.2016050005909err

**Published:** 2016-04-15

In “**Air pollution and its impacts on health in Vitoria, Espirito Santo, Brazil**”. Rev. Saude Publica [online]. 2016, vol. 50, 4. Epub 15-Mar-2016. ISSN 1518-8787. http://dx.doi.org/10.1590/S1518-8787.2016050005909, the Journal corrects the author’s last name.

**Where you read:**

Juger, Washington

**You should read:**

JUNGER, Washington

