# Supplementary material for: Air pollution and its impacts on health in Vitoria, Espirito Santo, Brazil
Source: Rev Saude Publica. 2016 Mar 1;50:4. doi: 10.1590/S1518-8787.2016050005909 (PMC4793970; doi:10.1590/S1518-8787.2016050005909)
Supplement: Translation

# Translation: Poluição do ar e impactos na saúde em Vitória, Espírito Santo

**Authors:** Clarice Umbelino de Freitas, Antonio Ponce de Leon, Washington Juger, Nelson Gouveia

**Affiliations:** I Laboratório de Investigação Médica. Hospital das Clínicas. Faculdade de Medicina. Universidade de São Paulo. São Paulo, SP, Brasil; II Instituto de Medicina Social. Universidade Estadual do Rio de Janeiro. Rio de Janeiro, RJ, Brasil; III Departamento de Medicina Preventiva. Faculdade de Medicina. Universidade de São Paulo. São Paulo, SP, Brasil

**Keywords:** Criança, Adulto, Poluição do Ar, efeitos adversos, Doenças Respiratórias, epidemiologia, Doenças Cardiovasculares, epidemiologia, Estudos de Séries Temporais

## Abstract

**OBJETIVO:**

Analisar o impacto da poluição atmosférica na morbidade respiratória e cardiovascular de crianças e adultos em Vitória.

**MÉTODOS:**

Foi realizado estudo utilizando modelos de séries temporais via regressão de Poisson a partir de dados de hospitalizações e poluentes em Vitória, ES, de 2001 a 2006. Foram testadas como variáveis independentes o material particulado fino (PM_10_); o dióxido de enxofre (SO_2_) e o ozônio (O_3_) em defasagem simples e acumulada até cinco dias. Introduziu-se temperatura, umidade e variáveis indicadoras dos dias da semana e feriados da cidade como variáveis de controle nos modelos.

**RESULTADOS:**

Para cada incremento de 10 µg/m^3^ dos poluentes PM_10_, SO_2_ e O_3_, foram observados aumentos no risco relativo percentual (RR%) para as hospitalizações por doenças respiratórias totais de 9,67 (IC95% 11,84-7,54), 6,98 (IC95% 9,98-4,17) e 1,93 (IC95% 2,95-0,93), respectivamente. Encontrou-se RR% = 6,60 (IC95% 9,53-3,75), RR% = 5,19 (IC95% 9,01-1,5) e RR% = 3,68 (IC95% 5,07-2,31) para doenças respiratórias em menores de cinco anos para o PM_10_, SO_2_ e O_3_, respectivamente. As doenças cardiovasculares apresentaram relação significativa com o O_3_ com RR% = 2,11 (IC95% 3,18-1,06).

**CONCLUSÕES:**

As doenças respiratórias apresentaram relação mais forte e consistente com os poluentes pesquisados em Vitória. Observou-se melhor relação dose-resposta quando se utilizou defasagens acumuladas em modelos de distribuição polinomial.

## INTRODUÇÃO

Os efeitos da poluição do ar na saúde são detectados em diversas cidades no mundo. Isso levou a Organização Mundial da Saúde (OMS) a propor metas de redução a partir de Padrões de Qualidade do Ar decrescentes, em substituição à regulamentação anterior, vigente até o ano de 2005^16^. Os estudos de impacto da poluição do ar na saúde nos países da América Latina chegam a resultados similares àqueles nas demais localidades no mundo^12^. A maior parte dos estudos se refere a poluentes atmosféricos decorrentes primária ou secundariamente da queima de combustíveis fósseis: material particulado fino (PM_10_), dióxido de enxofre (SO_2_), monóxido de carbono (CO), óxidos de nitrogênio (NO_x_) e ozônio (O_3_). Quanto aos desfechos de saúde, as doenças respiratórias e cardiovasculares são as mais comumente relacionadas à contaminação atmosférica^16^.

Vitória integra uma área geográfica de grande urbanização denominada região metropolitana da Grande Vitória. É a quarta cidade mais populosa do Espírito Santo (327.801 habitantes, numa área de 98 km^2^) segundo dados do Instituto Brasileiro de Geografia e Estatística (IBGE) de 2010. É cercada pela Baia de Vitória, uma ilha do tipo fluviomarinho. Possui dois portos que fazem parte do maior complexo portuário do Brasil (Porto de Vitória e Porto do Tubarão). A cidade conta com uma rede automática de monitoramento da qualidade do ar (RAMQAR) desde 2000. Essa RAMQAR tem três estações (Figura 1) e mede os parâmetros PM_10,_ SO_2_, O_3,_ CO e hidrocarbonetos nos bairros de Jardim Camburi, Enseada do Suá e Vitória Centro ^a^. A estação de Carapina, em município próximo de Vitória, além da mensuração do PM_10_, realiza o monitoramento de parâmetros meteorológicos.

**Figura 1:** Estações de monitoramento da qualidade do ar. Vitória, ES.

Grandes projetos industriais foram implantados na Região da Grande Vitória desde o início da década de 1970, e a indústria era apontada como a principal fonte de poluição da área^a^. De acordo com a mesma fonte, o crescimento da frota veicular e empreendimentos imobiliários têm alterado o perfil da região nos últimos anos. Inventário de fontes realizado pelo Instituto Estadual de Meio Ambiente e Recursos Hídricos (IEMA) em 2011 levanta a importância da ressuspensão de poeira (68,0%), que tem origens diversas, das indústrias (21,0%) e dos veículos (4,0%), como principais responsáveis pelos níveis de PM_10_. As emissões industriais foram responsáveis por quase toda a emissão de SO_2_ para a atmosfera e por mais da metade dos NOx, e teve pequena participação relativa para os hidrocarbonetos. Por sua vez, os veículos foram os principais responsáveis pelas emissões de CO e hidrocarbonetos (em torno de 50,0%)^b^. Dados do Portal Cidades do IBGE de 2005 indicam uma frota de 83.859 automóveis, com tendência crescente, chegando a 124.534 em 2014, de um total de 191.143 veículos em todas as modalidades^c^.

Publicações acerca dos efeitos da poluição atmosférica em várias cidades brasileiras utilizam abordagens diferentes entre si, o que não tem permitido comparações entre os achados. A metodologia utilizada neste estudo foi aplicada às diversas cidades brasileiras que contam com monitoramento da qualidade do ar, buscando dar subsídios para comparação entre os achados. O presente trabalho teve por objetivo estimar o risco de adoecimento por doenças respiratórias e cardiovasculares e sua relação com a poluição do ar em Vitória.

## MÉTODOS

Dados de internações hospitalares segundo local de residência foram obtidos do Sistema de Informações Hospitalares (SIH) do Datasus de 2001 a 2006 para o município de Vitória, a partir de um portal de informações construído para o Projeto de Avaliação de Impacto da Poluição do Ar nas Cidades Brasileiras. O projeto tinha como objetivos a mensuração do impacto da poluição do ar na saúde, utilizando a mesma metodologia para as diversas localidades, e a exploração de modelos de vigilância ambiental.

O portal refere-se às Autorizações de Internação Hospitalar (AIH) sistematizadas pelo Departamento de Informática do Sistema Único de Saúde (SUS), obtidos do Datasus. As bases foram agrupadas por dia, ou seja, contagens diárias de internações pelas causas investigadas: doenças respiratórias (CID10: J00-J99) em todas as idades (DRT); doenças respiratórias (CID10: J00-J99) em menores de cinco anos (DRC) e doenças cardiovasculares (CID10: I00-I99) em maiores de 39 anos (DCV), utilizadas como variáveis dependentes nos modelos. As variáveis de poluentes foram agregadas segundo estações de monitoramento e variáveis meteorológicas no mesmo portal. Os dados meteorológicos e de poluentes das três estações de monitoramento da cidade de Vitória, cedidos pelo IEMA, foram agregados em médias diárias para o PM_10_ e SO_2_ e a maior concentração de 8h para o O_3_, minimizando as perdas caso se considerassem as estações individualmente. As bases foram avaliadas quanto à continuidade das informações, aceitando-se perdas de no máximo 15,0% dos dias no período para cada variável ambiental. Os valores das concentrações de PM_10_, O_3_ e SO_2_ foram utilizados como variável de exposição e a temperatura e a umidade relativa do ar, como controles.

Tendo como fonte de informação os dados consolidados no portal, foi realizada análise de séries temporais com a biblioteca *ares* desenvolvida para o aplicativo R^d^, utilizando metodologia comum para as cidades que aderiram ao projeto. Para cada município participante, foram construídos modelos explicativos para contagens de internações pelas causas estudadas ao longo do tempo. Os modelos propostos pertenceram à classe de Modelos Aditivos Generalizados (GAM), com a opção de regressão de Poisson. Nessa classe de modelos, o número médio diário dos eventos de saúde era descrito como soma de funções das variáveis explicativas. Termos cuja associação com o desfecho era linear, por exemplo, dias da semana e feriados, entraram no modelo multiplicados por um coeficiente angular. Variáveis que se relacionaram com o desfecho de forma não linear entraram no modelo por meio de funções suaves, por exemplo, *splines*, segundo a equação:



em que *Y*_*t*_ *e X*_*1t*_ são os números de eventos mórbidos e o nível de um dado poluente no dia *t*, respectivamente; *X*_*it*_ são as variáveis preditoras, que inclui o tempo, e *S*_*i*_ são as funções de suavização, utilizando *natural splines*. Quando foram acrescentadas variáveis indicadoras para os dias da semana e feriados nacionais ou locais, testou-se sua significância. Os feriados com significância de até 0,09 foram agrupados segundo a direção do seu efeito: positiva ou negativa. No processo de modelagem da série temporal buscou-se minimizar o Critério de Informação de Akaike (AIC) e otimizar a Função de Auto Correlação Parcial (PACF).

Após a construção do modelo de trabalho (*Core Model)* contendo todas as variáveis de controle e da verificação de sua adequação, foram introduzidos, individualmente, os poluentes em defasagens de até cinco dias (*lag* simples). Foi analisado também o efeito cumulativo nesse período, a partir de um modelo polinomial de defasagens distribuídas. Esse modelo, além de considerar a latência do efeito dos poluentes, minimiza a instabilidade no processo de estimação, próprio das análises que utilizam múltiplas defasagens^13^.

O efeito foi estimado para cada poluente introduzido ao modelo de trabalho de forma linear, fornecendo o risco relativo percentual (RR%) para cada incremento de 10 μg/m^3^. Este é derivado a partir do RR, utilizando-se a seguinte fórmula: RR% = (*e* ^10 β^ - 1)*100. Essa quantidade expressa a variação percentual no número médio de internações hospitalares para a variação de 10 µg/m^3^ na concentração do poluente. Foi assumido o nível de significância de 5% em todas as estimativas.

## RESULTADOS

Foi encontrado menor número de internações em crianças, com média de duas internações por dia para doenças respiratórias. As perdas de dados meteorológicos e de poluentes estiveram abaixo de 15,0% de janeiro de 2001 a dezembro de 2006, limite estabelecido como aceitável para o período (Tabela 1).

**Tabela 1:** Parâmetros descritivos básicos. Vitória, ES, 2001-2006.

Parâmetro	Número de dias com dados	na	Média	DP	mín	máx	p_25_	p_50_	p_75_
Doenças respiratórias totais	2.191	0	3,9	2,4	0	15,0	2,0	4,0	5,0
Doenças respiratórias em < 5 anos	2.191	0	2,1	1,7	0	10,0	1,0	2,0	3,0
Doenças cardiovasculares em > 39 anos	2.191	0	3,7	2,5	0	15,0	2,0	30	5,0
PM_10_	2.164	27,0	27,1	7,0	6,5	61,0	22,5	26,67	31,0
O_3_	1.917	274,0	45,2	17,3	0	119,0	33,0	44,0	56,0
SO_2_	2.154	370	11,7	5,5	1,0	54,0	8,0	11,0	15,0
T (^o^C)	2.166	25,0	24,4	2,5	16,8	30,4	22,48	24,4	26,4
UR (%)	2.157	34,0	78,8	5,8	57,8	95,5	74,8	78,6	82,6

na: número de dias sem informação; PM_10_: material particulado menor que 10µ; O_3_: ozônio; SO_2_: dióxido de enxofre; T: temperatura em graus Celsius; UR: umidade relativa do ar

Apesar da sazonalidade característica dos poluentes, as médias anuais do PM_10_ mantiveram-se constantes e em torno de 27 µg/m^3^ no período. O SO_2_ apresentou tendência crescente, com níveis variando de 5,87 µg/m^3^ em 2001 a 14,06 µg/m^3^ em 2006. Os dados de ozônio apresentam descontinuidade entre 2005 e 2006, por problemas na estação de monitoramento, e não permitiram a observação de tendência (Figura 2).

**Figura 2:** Níveis diários de poluentes (PM10, SO2 e Ozônio). Vitória, ES, 2001-2006.

PM_10_: material particulado menor que 10µ; O_3_: ozônio; SO_2_: dióxido de enxofre

A análise de resíduos indicou bom ajuste do modelo para os desfechos pesquisados. Mesmo com o pequeno número de internações por doenças respiratórias em menores de cinco anos, a distribuição dos resíduos padronizados comparada à distribuição normal em quantis foi similar à distribuição para as doenças cardiovasculares e para todas as respiratórias.

Quando da introdução dos poluentes em defasagem simples nos modelos, foi encontrada relação significativa para o PM_10_ no lag 0 com RR% de 4,40 (IC95% 0,64-8,23), quando se analisaram as DRT. Foi encontrada relação no lag 2 para as DRC: RR% = 2,55 (IC95% 0,08-5,08) também para o O_3_. As doenças cardiovasculares não foram relacionadas a qualquer dos poluentes estudados nessa estrutura de defasagem. A Tabela 2 apresenta os RR% do modelo polinomial de defasagens distribuídas de zero a cinco dias para incrementos de 10 µg/m^3^ nos níveis de poluentes e seu valor global. O valor global alcançou níveis elevados no período para as internações por doenças respiratórias totais e em menores de cinco anos. As DCV relacionaram-se apenas com o ozônio.

**Tabela 2:** Risco Relativo percentual* e IC95% para internações por doenças respiratórias totais, doenças respiratórias em menores de cinco anos e doenças cardiovasculares em maiores de 39 anos. Vitória, ES, 2001-2006.

Poluente	Defasagem	DAR Totais			DAR < 5 anos			DCV > 39 anos		
		
		RR%	IC95%		RR%	IC95%		RR%	IC95%	
PM_10_	Dia corrente	1,52	-1,85	5,02	0,27	-4,34	5,09	-1,55	-4,87	1,88
	Defasagem de 1 dia	2,11	0,12	4,13	1,43	-1,28	4,22	-0,14	-2,12	1,88
	Defasagem de 2 dias	2,26	0,37	4,18	1,97	-0,62	4,62	0,66	-1,23	2,6
	Defasagem de 2 dias	1,98	0,15	3,85	1,87	-0,65	4,45	0,84	-1,02	2,75
	Defasagem de 2 dias	1,28	-0,43	3,03	1,13	-1,23	3,56	0,39	-1,39	2,2
	Defasagem de 2 dias	0,16	-2,74	3,16	-0,22	-4,23	3,95	-0,68	-3,62	2,35
	Acumulado 5 dias	9,67	7,54	11,84	6,60	3,75	9,53	-0,49	-2,46	1,52
O_3_	Dia corrente	-0,50	-2,32	1,34	0,33	-2,13	2,86	1,03	-0,88	2,98
	Defasagem de 1 dia	0,08	-0,91	1,07	0,84	-0,49	2,2	0,26	-0,77	1,3
	Defasagem de 2 dias	0,47	-0,49	1,43	1,06	-0,23	2,36	-0,14	-1,13	0,87
	Defasagem de 2 dias	0,67	-0,27	1,63	0,96	-0,32	2,26	-0,16	-1,14	0,83
	Defasagem de 2 dias	0,69	-0,24	1,63	0,57	-0,68	1,83	0,19	-0,76	1,16
	Defasagem de 2 dias	0,52	-1,19	2,25	-0,13	-2,41	2,21	0,92	-0,86	2,73
	Acumulado 5 dias	1,93	0,93	2,95	3,68	2,31	5,07	2,11	1,06	3,18
SO_2_	Dia corrente	1,36	-3,4	6,35	-0,25	-6,53	6,44	-5,21	-9,88	-0,31
	Defasagem de 1 dia	0,20	-2,44	2,91	0,55	-2,98	4,2	-2,1	-4,83	0,72
	Defasagem de 2 dias	-0,15	-2,74	2,5	1,08	-2,38	4,66	0,37	-2,39	3,2
	Defasagem de 2 dias	0,29	-2,29	2,94	1,34	-2,11	4,91	2,13	-0,65	4,98
	Defasagem de 2 dias	1,53	-0,97	4,09	1,33	-1,99	4,76	3,14	0,45	5,9
	Defasagem de 2 dias	3,61	-0,87	8,29	1,04	-4,81	7,25	3,38	-1,34	8,33
	Acumulado 5 dias	6,98	4,17	9,88	5,19	1,5	9,01	1,42	-1,41	4,34

DAR Totais: Internações por doenças respiratórias totais; DAR < 5 anos: Internações por doenças respiratórias em menores de cinco anos; DCV > 39 anos: Internações por doenças cardiovasculares em maiores de 39 anos; PM_10_: material particulado menor que 10µ; O_3_: ozônio; SO_2_: dióxido de enxofre

* Expressa a variação percentual no número médio diário de internações hospitalares para uma variação de 10 µg/m^3^ na concentração do poluente.

## DISCUSSÃO

Os desfechos de saúde apresentaram relação mais robusta e constante quando se consideraram os efeitos acumulados dos poluentes de zero a cinco dias em Vitória. As internações por doenças respiratórias totais e doenças respiratórias em menores de cinco anos foram relacionadas a todos os poluentes pesquisados, com maior força de associação com o PM_10_. As internações por doenças cardiovasculares estiveram associadas apenas às variações nos níveis de ozônio. Os resultados expressos em RR% traduzem o quanto aumenta o número médio diário de internações para cada incremento de 10 µg/m^3^ do poluente.

Os níveis de PM_10_ (27 µg/m^3^) em Vitória de 2001 a 2006 mantiveram-se abaixo dos Padrões de Qualidade do ar de média anual propostos pelo Conselho Nacional de Meio Ambiente (CONAMA) de 50 µg/m^3^, e próximos da recomendação guia da OMS de 20 µg/m^3^. Apesar disso, foi encontrado efeito da poluição do ar nos moradores de Vitória. Isso corrobora as recomendações da OMS de que se deve buscar sempre os menores níveis de exposição para proteção da saúde, mesmo em cidades pouco poluídas, visto que a relação dose resposta é linear quando se analisam os efeitos dos contaminantes atmosféricos, sem dose segura^4^ ^,^ ^16^. Vitória possui característica mista de exposição (indústria e veículos contribuem com os níveis e de poluentes) e deve ter composição de PM_10_ diferente de outros centros urbanos onde predominam veículos. O entendimento dos efeitos de cada tipo de fonte de exposição passa por estudos de especiação do material particulado, não disponíveis na localidade.

Embora se tenha considerado para estudo um período com maior completude de dados, observou-se lacuna nas informações dos níveis de ozônio. Essas lacunas, apesar de dentro de limites toleráveis segundo os critérios estabelecidos pelos autores, concentram-se em um único período de 2005, o que pode ter influenciado os resultados das análises desse poluente.

Dentre os poluentes, o PM_10_ apresenta relação mais frequente e consistente com as doenças do aparelho respiratório e cardiovascular, embora nem sempre seja possível separar os efeitos dos demais poluentes, uma vez que todos estão presentes no ar que respiramos^1^. Estudo de revisão sobre os mecanismos de ação do PM_10_ aponta para o desencadeamento de *stress* oxidativo e processo inflamatório sistêmico que podem estar relacionados ao remodelamento anatômico e fisiológico do pulmão e com o processo de aterosclerose^1^. Encontramos relação entre as doenças respiratórias e o PM_10_ em Vitória. Esse poluente não esteve relacionado às internações por doenças cardiovasculares.

Bell et al.^2^ realizaram metanálise de estudos de séries temporais e encontraram relação entre ozônio e mortalidade geral e por doenças cardiovasculares, quando consideradas cidades americanas e de outras localidades. As mortes por doenças respiratórias não foram associadas aos níveis de ozônio. Em recente publicação de 34 estudos avaliando os efeitos do PM_10_, PM_2,5_, CO, NO_2_, SO_2_ e O_3_ no infarto do miocárdio, não foi encontrada relação significativa entre os níveis do último poluente e este desfecho^10^. Foi encontrada associação significativa entre exposição ao ozônio e internações por doenças respiratórias em crianças^8^ ^,^ ^11^ e cardiovasculares em maiores de 39 anos residentes em Cubatão, SP^11^. Em São Paulo, SP, essa relação foi observada para doenças respiratórias em crianças, com força de associação menor que a do presente estudo. Em São Paulo, avaliou-se o risco de internações para incrementos de 10 µg/m^3^, o que permitiu comparação com nossos achados^5^.

A literatura mostra que os efeitos na saúde continuam a ser detectados mesmo em baixas exposições a dióxido de enxofre^15^. Isso deve constituir-se em preocupação maior em Vitória, uma vez que se observou tendência de aumento desse poluente no período analisado. Essa tendência persistiu, como referido no Relatório Anual de Qualidade do Ar de Vitória de 2013 em análise dos últimos 10 anos.

Em levantamento da literatura sobre efeitos da poluição do ar na saúde dos residentes de Vitória, foram encontradas três publicações. A primeira^9^ explora a proporção atribuível de óbitos por doenças respiratórias em idosos e crianças na população urbana do Brasil, incluindo Vitória, a partir da aplicação do coeficiente de relação dose-resposta de estudos de séries temporais realizados no Rio de Janeiro, RJ, e São Paulo. A segunda^3^ analisa as taxas de atendimento ambulatorial por asma segundo bairros de Vitória. A terceira^14^ explora modelos de séries temporais, de um lado aqueles já consagrados na literatura e de outro, utilizando a análise de componentes principais. Os autores buscam estabelecer a relação dose resposta entre poluentes e internações em crianças a partir das duas abordagens, apontando para melhor adequação do modelo e do impacto do poluente quando se utiliza a análise de componentes principais. Com essa abordagem, foi encontrado RR% = 3,00 para o incremento de 10,49 µg/m^3^ de PM_10_, enquanto o RR% obtido a partir da abordagem convencional foi de 2,00 para o mesmo incremento do poluente. Embora seja difícil a comparação quando se utiliza metodologia de análise diversa, o presente estudo chegou a estimativa mais elevada para crianças menores de cinco anos, com RR% = 6,6 para cada incremento de 10 µg/m^3^ de PM_10_, quando considerado o efeito acumulado de zero a cinco dias.

O sistema de informações das AIH não representa a totalidade de hospitalizações do Brasil. Refere-se aos serviços pagos pelo Sistema Único de Saúde e sua cobertura é variável entre os estados da federação. O sistema de informações da Comunicação de Internação Hospitalar (CIH), que conta com o registro de internações dos hospitais não conveniados ao SUS, está disponível no Datasus a partir de 2008. As CIH corresponderam a 16,9% das hospitalizações em Vitória em 2008. Mantendo-se esta proporção, o presente estudo pode ter utilizado mais de 80,0% das hospitalizações da cidade. No entanto, deve-se ter cautela ao interpretar os resultados. Eles devem ser considerados como o impacto da poluição do ar nas internações registradas nos hospitais conveniados ao SUS.

Os estudos que visam medir o impacto da poluição na saúde são uma ferramenta dos técnicos de Vigilância em Saúde para discutir atividades de prevenção e controle com órgãos regulamentadores ou com o poder judiciário^6^. Por um lado, o setor saúde conta com pouca competência legal para exigir dos poluidores medidas que visem corrigir a contaminação do meio ambiente. Por outro, conta com instrumentos que lhe permitem medir os danos provocados por essa contaminação na população, embora com técnicas de aferição complexas, nem sempre acessíveis aos serviços de atenção básica.

A avaliação e acompanhamento dos danos à saúde provocados pela contaminação do ar utilizando estudos de séries temporais é proposta como atividade de vigilância pelo Ministério da Saúde^7^. Em estudo recente^7^, o PM_10_ foi proposto como indicador de exposição e as internações por doenças respiratórias como indicador de efeito para fins de vigilância dos impactos da poluição do ar na saúde nas cidades brasileiras. Uma vez que o setor saúde não dispõe de mecanismos legais para o controle da poluição atmosférica, sua contribuição no estabelecimento de políticas passa pela quantificação do impacto da poluição na carga de doenças da população. Os estudos de séries temporais, embora medindo apenas os efeitos agudos da poluição do ar, podem ser utilizados para a conscientização dos gestores e das pessoas corroborando a adoção de políticas que visem a melhoria do ar que respiramos. A área ambiental de Vitória pode utilizar esses estudos para avançar nas políticas de controle da qualidade do ar da cidade. O estímulo à continuidade e diversificação de abordagens que visem relacionar as condições ambientais adversas a seus impactos na saúde é não só desejável, mas necessário para a estruturação tanto da Saúde Ambiental no Brasil como da América Latina como um todo.

## Footnotes

a Instituto Estadual de Meio Ambiente e Recursos Hídricos. Relatório da qualidade do ar: Grande Vitória: 2013 [Internet]. Vitória (ES): Instituto Estadual de Meio Ambiente e Recursos Hídricos; [citado 2014 set 14]. Disponível em: http://www.meioambiente.es.gov.br/download/Relat%C3%B3rio_Anual_de_Qualidade_do_Ar_2013.pdf

b Instituto Estadual de Meio Ambiente e Recursos Hídricos. Meio ambiente: estudo mostra como está a qualidade do ar na GV [Internet]. *Diário Oficial de Poderes de Estado*. Vitória (ES) 11 maio 2011, 3. [citado 2015 nov 26]. Disponível em: file:///C:/Users/Note/Downloads/diario_oficial_2011-05-11_completo.pdf

c Instituto Brasileira de Geografia e Estatística, Cidades. Espírito Santo: Vitória. Rio de Janeiro (RJ): Instituto Brasileira de Geografia e Estatística; 2015 [citado 27 nov 2015]. Disponível em: http://www.cidades.ibge.gov.br/xtras/perfil.php?lang=&codmun=320530

d JungerWL. Análise, imputação de dados e interfaces computacionais em estudos de séries temporais epidemiológicas [tese]. Rio de Janeiro (RJ): Universidade Estadual do Rio de Janeiro; 2008.
